# An In Vitro Validation of DIAGNOdent Versus Radiography for the Detection of Caries Underneath Different Types of Restorations

**DOI:** 10.7759/cureus.63689

**Published:** 2024-07-02

**Authors:** Osama S Mohamed, Salma I Almaz, Nouran R Moustafa, Ahmed A Ibrahim, Mohamed A Hall, Inas Karawia

**Affiliations:** 1 Dental Prosthesis Manufacture Technology Department, Faculty of Applied Health Sciences Technology, Pharos University in Alexandria, Alexandria, EGY; 2 Faculty of Dentistry, Pharos University in Alexandria, Alexandria, EGY; 3 Alexandria Dental Research Center, Ministry of Health and Population, Alexandria, EGY; 4 Pediatric and Community Dentistry Department, Faculty of Dentistry, Pharos University in Alexandria, Alexandria, EGY

**Keywords:** diagnodent, dental caries, diagnosis, secondary caries under restoration, radiographic examination

## Abstract

Objective

This study aimed to assess the validity of DIAGNOdent as a diagnostic device for the detection of secondary dental caries underneath different types of restorations.

Methods

A total of 60 extracted human permanent molars were used in this experimental study (30 sound teeth and 30 teeth with proximal caries); 20 teeth (10 sound and 10 carious) were randomly allocated into one of three restoration groups: composite, amalgam, or resin-modified glass ionomer (RMGI). All teeth were examined using both KaVo DIAGNOdent Classic and digital radiographic sensors.

Results

In the composite-restored teeth, DIAGNOdent showed excellent sensitivity (90%) and specificity (90%). On the other hand, digital radiography had high sensitivity (80%) and poor specificity (20%). In amalgam-restored teeth, DIAGNOdent showed low sensitivity (30%) and excellent specificity (100%), while radiographs showed good sensitivity (70%) and low specificity (30%). In the RMGI-restored teeth, DIAGNOdent had excellent sensitivity (100%) and 70% specificity, while digital radiography had poor sensitivity (30%) and excellent specificity (90%).

Conclusions

DIAGNOdent showed superior results in detecting secondary caries lesions underneath composite and RMGI restorations when compared to digital radiography. On the other hand, radiography emerged as a preferable method for the detection of secondary caries underneath amalgam restorations. Based on our findings, DIAGNOdent can be used in dental clinics for the early diagnosis of hidden secondary caries underneath composite and RMGI restorations.

## Introduction

Dental caries, including a high incidence of secondary caries, continues to be one of the most common diseases in the world [1,2], despite the considerable recent advancements in preventative dentistry and the increasing quality of restorative materials. Secondary caries can be difficult to identify because dental tissue underneath restorations is not directly examined [3]. For this reason, dentists have been seeking a reliable test or tool to identify secondary caries beneath restorations [4].

The validity of a diagnostic device is judged based on its ability to differentiate between individuals with and without a disease. The two important measures of validity are sensitivity and specificity. Sensitivity is defined as the degree to which a particular test correctly identifies sick individuals, while specificity is the test’s ability to detect when individuals do not have a disease. Diagnostic devices should not be used until their accuracy is fully ensured, as most diagnostic devices are not always perfect or accurate [5]. Among the currently available tools for the diagnosis of secondary caries, analog or digital radiography is used to examine dental caries underneath restorations. However, several factors may influence radiographic interpretation, including the clinician’s capabilities, the size and location of the carious lesion, and exposure parameters. Furthermore, composite restoration porosity might lead to false-positive outcomes. Hence, there is an urgent need for a new method for accurately detecting caries underneath restorations without requiring an expert operator [3].

DIAGNOdent is a non-invasive laser fluorescence diagnostic tool that evaluates the difference in fluorescence between normal and carious dental tissue, thereby avoiding exposure to harmful radiation. Additionally, it reduces human error by assigning a different score to each tissue [6]. DIAGNOdent was first introduced to the market by KaVo, a German company, in 1998 [7], to be used alongside visual examination to diagnose occlusal caries. Its decay detection involves the use of a diode laser with a wavelength of 655 nm pointed at a dental surface, which is then absorbed by the intraoral bacterial byproducts that emit red fluorescence. The fluorescence reflected off the dental surface is displayed on the device’s screen as a number ranging from 0 to 99. The greater the number, the deeper the carious lesion. Laser fluorescence offers a non-invasive quantitative method for diagnosing dental caries [8,9].

A study conducted in 2016 used histologic assessment to compare the accuracy of radiography and DIAGNOdent in identifying secondary caries underneath restorations. The results demonstrated that radiographs are not as accurate as the DIAGNOdent device in identifying caries underneath restorations, indicating that DIAGNOdent is an effective method for identifying carious cavities under composite restorations [3]. Furthermore, Abrams et al. investigated the efficacy of four different modalities for detecting caries around resin-modified glass ionomer (RMGI) [10] and amalgam restorations [11]. They concluded that while DIAGNOdent can differentiate between healthy and carious tissue in restoration margins, it has greater variation and less reliability than other methods.

The majority of the extant studies have examined the efficacy of DIAGNOdent in diagnosing secondary dental caries around restoration or in margins, which can be detected visually [3,10]. However, the effectiveness of DIAGNOdent in assessing hidden caries beneath dental restorations is not clear due to conflicting findings and a general paucity of studies. Furthermore, variability in restoration types and depths of caries has complicated the understanding of DIAGNOdent’s reliability. In light of this, we conducted this study to fill the existing knowledge gap, by evaluating the diagnostic validity of DIAGNOdent in comparison with digital radiography in detecting dental caries underneath three types of restorations: composite, amalgam, and RMGI, under standardized conditions.

## Materials and methods

An in vitro, prospective, comparative diagnostic accuracy study was conducted from August 2023 to December 2023. The minimum required sample size was calculated using G-power software version 3.1.9.4 with a 2.2 effect size as per t-test based on a previous study [2], using 0.5 alpha error, and 95% confidence interval with a power of 98%, involving 20 extracted human molars for each restoration (60 teeth in total for the three restorations). Approval was obtained from the Research Ethics Committee at Pharos University in Alexandria, Egypt (approval no: UREAC-04-3-203). Teeth were collected from the dental clinics at Pharos University, as well as public and private clinics in Alexandria. The study followed the Standards for Reporting of Diagnostic Accuracy Studies (STRAD) guidelines [12].

Thirty freshly extracted sound molars and another 30 molars with proximal active caries lesions [11] were collected. Teeth with severe crown destruction, heavily restored teeth, badly decayed teeth in which caries reached half of the proximal surface, pulpally involved, and root canal-treated teeth were excluded. Scaling was undertaken using the ultrasonic scaler (Cavitron, Dentsply, York, PA), followed by polishing using prophypaste with polishing brushes. Teeth were subsequently preserved in normal saline to ensure no effect on DIAGNOdent readings. Twenty teeth (10 sound teeth and 10 carious) were randomly allocated to each type of restoration: composite (Meta Biomed Nexcomp composite shades A3 for dentin and A2 for enamel), amalgam (Admixed high copper amalgam capsules), and RMGI (Riva light cure resin-modified GI cement). All teeth were inserted into small wax blocks to ease their handling during restoration and finishing. Then, teeth were randomly coded with a number from 1 to 60, to ensure blindness and hence no bias on the part of the researcher examining the sample with DIAGNOdent.

Standardized class II cavity preparation was performed on all teeth by a researcher using diamond burs (SF-14, Dian Fong, Shenzhen, China). The dimensions of the cavities were 4 mm width bucco-lingually, and the gingival step, for receiving one of the three restorations, was located 1 mm above the cement-enamel junction. In the carious teeth, residual caries was intentionally left in the gingival step away from the intact margins, and clearing of the peripheral edge of the gingival step was conducted to ensure intact margins and guarantee blindness. One researcher prepared all the cavities and restorations. Standardization was done to ensure the same thickness of restoration materials in carious and sound molar teeth.

Radiographic examination

Radiographs of each tooth were taken in the wax block using Portable TDS Eighteeth X-ray radiography and a dental sensor with exposure settings at mA = 1 and Kvp = 70 for 0.1 seconds. The tube head and retentive arm length were set so that the tube had a fixed distance from the sensor. Radiographs of all teeth were documented with their codes on the software. The radiographic interpretation was performed by a staff member with 25 years of experience in the Dental Restorative Department, Faculty of Dentistry, Pharos University. The interpretation was repeated for all samples after one month to evaluate the intra-examiner reliability. 

DIAGNOdent examination

To imitate proximal contact in the oral cavity through contact with neighboring sound teeth, teeth were removed from the wax blocks and inserted in models with the same codes. DIAGNOdent was used according to the manufacturer’s instructions [6], with tip B attached to the device for smooth surface examination. The laser probe was moved from the buccal towards the lingual side beneath the contact area and from the lingual toward the buccal side without pressure. This procedure was repeated five times on each side and the highest peak value was registered. The peak score from 0-14 was considered sound and 14-99 was considered carious [3]. All molar teeth were decoded from their numeric format to recognize carious and sound teeth under each type of restoration. The existing knowledge regarding the condition of the teeth served as a benchmark for comparing the DIAGNOdent and radiograph.

Statistical analysis

After collection, the data were revised, coded, and analyzed using the statistical software IBM SPSS Statistics version 25 (IBM Corp., Armonk, NY). Sensitivity, specificity, positive predictive value (PPV), negative predictive value (NPV), accuracy parameters, and the area under the receiver operating characteristic (ROC) curve and area under the curve (AUC) were studied to assess the diagnostic performance of DIAGNOdent.

## Results

The Kappa test result was 0.8, indicating perfect agreement of intra-examiner reliability. When diagnosing caries underneath composite restorations, digital radiography demonstrated high sensitivity (80% of 10), low specificity (20% of 10), and fair PPV, NPV, and accuracy (50% of 20) compared to DIAGNOdent’s excellent sensitivity (90% of 10), specificity (90% of 10), PPV (90% of 10), NPV (90% of 10), and accuracy (90% of 20). The higher sensitivity, specificity, PPV, NPV, and accuracy showed that DIAGNOdent is more accurate than radiography for detecting caries underneath composite restorations (Table 1).

**Table 1:** Accuracy measures relating to DIAGNOdent and digital radiography in diagnosing caries under composite restoration

Accuracy measures	Total n	DIAGNOdent	Total n	Digital radiography
Sensitivity	10	90%	10	80%
Specificity	10	90%	10	20%
Positive predictive value (PPV)	10	90%	16	50%
Negative predictive value (NPV)	10	90%	4	50%
Accuracy	20	90%	20	50%

Underneath amalgam restoration, DIAGNOdent showed low sensitivity (30% of 10), excellent specificity (100% of 10), excellent PPV (100% of 3), fair NPV (58.8% of 17), and good accuracy (65% of 20) for diagnosing caries, while digital radiography had good sensitivity (70% of 10), low specificity (30% of 10), and fair PPV (50% of 14), NPV (50% of 6), and accuracy (50% of 20) for identifying caries in teeth (Table 2). DIAGNOdent had higher specificity, PPV, NPV, and accuracy for secondary caries diagnosis in teeth restored with amalgam, while radiography had higher sensitivity.

**Table 2:** Accuracy measures relating to DIAGNOdent and Digital radiography in diagnosing caries under amalgam restoration

Accuracy measures	Total n	DIAGNOdent	Total n	Digital radiography
Sensitivity	10	30%	10	70%
Specificity	10	100%	10	30%
Positive predictive value (PPV)	3	100%	14	50%
Negative predictive value (NPV)	17	58.8%	6	50%
Accuracy	20	65%	20	50%

For identification of caries under RMGI restoration, DIAGNOdent showed excellent sensitivity (100% of 10), good specificity (70% of 10), good PPV (75% of 13), excellent NPV (100% of 7), and high accuracy (84.2% of 20), while digital radiography had poor sensitivity (30% of 10), excellent specificity (90% of 10) and PPV (83.3% of 4), and good NPV (69.2% of 16) and accuracy (73.7% of 20) (Table 3). DIAGNOdent had higher sensitivity, NPV, and accuracy for diagnosing caries with RMGI restoration, while digital radiography had higher specificity and PPV.

**Table 3:** Accuracy measures relating to DIAGNOdent and digital radiography in diagnosing caries under resin-modified glass ionomer restoration

Accuracy measures	Total n	DIAGNOdent	Total n	Digital radiography
Sensitivity	10	100%	10	30%
Specificity	10	70%	10	90%
Positive predictive value (PPV)	13	75%	4	83.3%
Negative predictive value (NPV)	7	100%	16	69.2%
Accuracy	20	84.2%	20	73.7%

**Figure 1:**
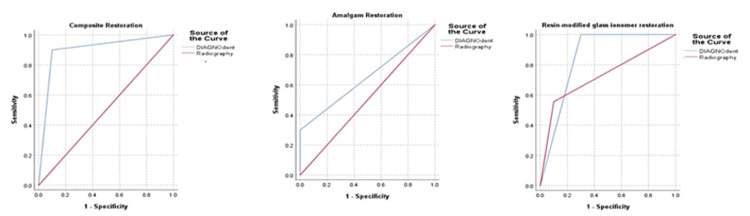
Difference in the ROC curves for the comparison between DIAGNOdent and digital radiography for the detection of caries in teeth under different restorations ROC: receiver operating characteristic

The final step involved comparing the data received from DIAGNOdent and digital radiography to the existing knowledge regarding the condition of the teeth. In the case of composite restoration, DIAGNOdent showed statistically significant higher diagnostic ability in caries detection, as evidenced by the AUC (0.9) and 95% CI (0.744-1.00). On the other hand, for amalgam restoration, the AUC was higher for DIAGNOdent (0.65), at 95% CI (0.403-0.897) than radiography (0.50; 0.241-0.759); however, there was no statistical significance (p-value: 0.257 and 1.0 respectively) regarding secondary caries detection beneath amalgam restoration. In the case of RMGI, DIAGNOdent showed a high AUC (0.85), at 95% CI (0.663-1.00), with statistically significant diagnostic ability in caries detection (p = 0.01) (Table 4; Figure 1).

**Table 4:** Comparison between DIAGNOdent and digital radiography in the diagnosis of caries under different restorations *Statistically significant AUC: area under the curve; CI: confidence interval

	Composite		Amalgam		Resin-modified glass ionomer	
	DIAGNOdent	Radiography	DIAGNOdent	Radiography	DIAGNOdent	Radiography
AUC (p-value)	0.90 (0.002^*^)	0.50 (1.0)	0.65 (0.257)	0.50 (1.0)	0.85 (0.01^*^)	0.728 (0.094)
95% CI	0.744-1.00	0.241-0.759	0.403-0.897	0.241-0.759	0.663-1.00	0.488- 0.968

## Discussion

Dental caries must be accurately diagnosed as early as possible to avoid its further progression [13]. Compared to primary caries detection, hidden secondary caries diagnosis underneath different restorations has proven more difficult. Several methods, including laser fluorescence, and digital radiography, have been used to identify secondary dental caries underneath restorations [14]. A preferred approach for caries detection would involve a device that does not demand specialized skills or extensive experience while delivering accurate and reliable performance. However, accurate diagnosis of secondary caries via radiography typically necessitates clinical expertise [15]. The DIAGNOdent device relies on detecting differences in fluorescence levels between healthy and carious dental tissues. Unlike radiography, DIAGNOdent does not emit ionizing radiation or require cooperation from the patient [16].

Diagnostic devices for dental caries should have accuracy and reliability, which rely on several variables. One key variable is a high level of sensitivity, which minimizes false-negative results [17]. Additionally, diagnostic devices should have high specificity, to avoid false-positive results of caries lesions and thereby prevent unneeded interventions [18]. The association between sensitivity and specificity is explained by the ROC, which evaluates the diagnostic accuracy of the examined device. The assessment of the evaluated device’s diagnostic interpretation is provided by the AUC [19].

The current study aimed to examine the accuracy of the DIAGNOdent device concerning secondary caries detection in extracted human teeth underneath various restorations (composite, amalgam, and RMGI) versus digital radiography. When the teeth were restored with composite, the results indicated that the performance of DIAGNOdent was better than radiography in all metrics of diagnostic accuracy. However, regarding caries identification under amalgam restoration, DIAGNOdent exhibited lower sensitivity than digital radiography, but higher specificity, PPV, and NPV than radiography. In contrast, when teeth were restored with RMGI, DIAGNOdent showed higher sensitivity, NPV, and accuracy than digital radiography. These results validate the accuracy of DIAGNOdent in the detection of secondary caries under composite and RMGI. In comparison with radiography, DIAGNOdent performed better, as evidenced by substantially higher AUCs for composite (0.90) and RMGI (0.85) restorations. However, the DIAGNOdent AUC for amalgam (0.65) was not statistically significant when compared with digital radiography.

Sichani et al. (2016) evaluated the accuracy of DIAGNOdent and radiography using histologic assessment for the detection of caries lesions under composite restorations in primary teeth. Consistent with our results, they found that DIAGNOdent has higher accuracy in caries detection than radiography (p = 0.0001), as well as higher sensitivity (70.97), specificity (83.72), PPV (75.9), NPV (80), and AUC (0.70) [3]. Bonding materials applied to the cavity floor underneath composite restorations can interfere with the proper radiographic diagnosis of secondary caries and may lead to misdiagnosis, especially if deposited in a thick layer. This could explain DIAGNOdent’s superior accuracy when compared to digital radiography. DIAGNOdent's efficiency in the identification of secondary proximal caries next to composite restorations was validated by evaluations of the diagnostic accuracy of DIAGNOdent and digital radiography. However, the superior performance of DIAGNOdent’s diagnostic performance was not statistically significant [2,20]. 

Some studies have highlighted that DIAGNOdent enhances the detection of secondary caries adjacent to amalgam restorations compared to radiography [21,22]. On the other hand, a recommendation against using DIAGNOdent for the detection of residual caries under amalgam restorations has been reported [21]. This suggests that a variety of circumstances may impact DIAGNOdent’s efficacy in diagnosing dental caries underneath amalgam restoration; mainly, the laser light’s access may be obstructed by the amalgam, as it is a metal alloy. Hence, the detection of deep carious lesions through clinically intact margins at a distance from the amalgam is considered difficult when using DIAGNOdent [11]. This explains the high specificity of DIAGNOdent in the current study, which was likely due to the inability for fluorescence penetration instead of detection of the sound teeth, and the good sensitivity of radiography in detecting caries underneath amalgam. However, in a radiograph, amalgam’s opacity makes translucent dental caries more apparent. Also, amalgam restoration has shown high radio-opacity on radiographs, which makes the radiolucent dental caries beneath more apparent due to the improved contrast [20,23].

DIAGNOdent's performance in the diagnosis of dental caries underneath composite and RMGI may be due to the translucency of both materials, which allows for the penetration of laser fluorescence. This would also explain the higher sensitivity of RMGI, which has less filler content and is more translucent than composite [24]. Caries underneath restorations can be exceedingly difficult to detect; DIAGNOdent can be utilized to identify such conditions [25]. Although it appears that laser light can penetrate composites and detect carious cavities beneath them, some variables might influence the device’s results and should therefore be considered [26], like polishing the restoration to remove any stains before measurement in order to avoid false readings [27]. Furthermore, variables identified in in vitro studies must be taken into account; e.g., antiseptic solutions can alter the fluorescence response and cause structural alterations in the tooth [28].

Based on the evidence, care should be taken to distinguish between laboratory and clinical settings. Clinical investigations are recommended to confirm DIAGNOdent’s superior ability in the diagnosis of caries [29]. In the current study, when molars were restored using composite materials, DIAGNOdent was shown to be a successful technique for identifying secondary caries. It is preferable to use DIAGNOdent with conventional diagnostic methods, such as radiography and clinical assessments, particularly when dealing with amalgam restorations [30].

While the current study has demonstrated some strengths, especially in assessing the DIAGNOdent's accuracy in detecting caries under three of the most commonly used restorations under controlled conditions such as blindness, standardization of methods, and an attempt to replicate tooth-to-tooth contact during examination with the DIAGNOdent, it has some limitations as well. In vitro studies cannot accurately represent the oral environment in real life. Hence, we recommend in vivo clinical studies to comprehensively assess DIAGNOdent's accuracy in diagnosing secondary caries beneath various restorations.

## Conclusions

Based on our findings, DIAGNOdent showed superior efficacy in secondary caries detection in comparison with digital radiography, especially underneath composite and RMGI restorations. Radiography showed better performance than DIAGNOdent in detecting caries underneath amalgam restorations. For better results in terms of treatment outcomes and cost-efficiency, DIAGNOdent is recommended for diagnosing and detecting secondary dental caries beneath composite and RMGI restorations, as well as for diagnosing early carious lesions. However, using DIAGNOdent for diagnosing secondary caries lesions underneath amalgam restorations is not recommended due to the high opacity of amalgam and the fact that it commonly consists of silver and mercury, which prevent the passage of the DIAGNOdent laser.
